# Risk of osteoporosis in patients with chronic inflammatory neuropathy- a population-based cohort study

**DOI:** 10.1038/s41598-019-45591-4

**Published:** 2019-06-24

**Authors:** Seung Woo Kim, Eun Hwa Kim, Jinae Lee, Young-Chul Choi, Seung Min Kim, Ha Young Shin

**Affiliations:** 1 Department of Neurology, Yonsei University College of Medicine, Seoul, South Korea; 2 Biostatistics Collaboration Unit, Department of Biomedical Systems Informatics, Yonsei University College of Medicine, Seoul, South Korea

**Keywords:** Neuromuscular disease, Peripheral neuropathies

## Abstract

The risk of osteoporosis in patients with chronic inflammatory neuropathy (CIN) has not been evaluated in detail. We conducted a population-based case-control study nested in a retrospective cohort to analyze osteoporosis risk among patients with CIN using a nationwide database. Patients with CIN based on the Korean Classification of Disease diagnostic code were included and were matched to controls. A Cox proportional hazards regression model was used to evaluate the effect of CIN on osteoporosis. After propensity score matching, 585 CIN patients and 585 controls were selected. Patients with CIN had an increased osteoporosis risk (hazard ratio [HR] = 2.293, 95% confidence interval [CI] 1.460–3.601) compared with controls. The osteoporosis risk was higher among male patients with CIN than among male controls (HR = 5.404, 95% CI 2.252–12.969), while there were no significant differences among women. Among the CIN patients, the average daily dose of corticosteroids was higher in those who developed osteoporosis (19.6 mg [10.8–49.3]) than those who did not (16.2 mg [7.2–29.1], p = 0.001). The osteoporosis risk among CIN patients is higher than among controls. High risk of osteoporosis in male patients may indicate that osteoporosis in CIN patients results from the disease itself or related treatments.

## Introduction

Chronic inflammatory neuropathy (CIN), which includes chronic inflammatory demyelinating polyradiculoneuropathy (CIDP) and multifocal motor neuropathy (MMN), is a rare immune-mediated disease of the peripheral nerves that shows a progressive clinical course^1–3^. Patients with CIDP often develop substantial disability and nearly 20 to 30% of patients experience a functional status of being wheelchair bound or worse^4,5^. Although not as severe as in CIDP, MMN can also present with lower limb weakness and more than half of patients display abnormal gait before treatment^6^. Recently, we revealed that the bone mass of patients with CIDP was significantly lower than that of normal controls and low bone density was associated with impaired functional status^7^. Reduced bone mass, along with lower limb weakness and gait unsteadiness^8^, can increase the risk of fracture in patients with CIN.

It is important to assess the risk of osteoporosis or fracture in patients with CIN. A previous report revealed that over half of patients with CIDP developed osteoporosis within 3 years^9^. However, only a few studies with small study populations have assessed the risk of osteoporosis among patients with CIN, and there is a lack of comprehensive analyses of the effect of each risk factor on osteoporosis. This could be due to the low prevalence of CIN, which ranges from 1 to 4.8 per 100,000 for CIDP and 1–2 per 100,000 for MMN^10–14^. In order to overcome this limitation, a cohort study based on a large study population is required. Thus, we analyzed the occurrence of osteoporosis in patients with CIN using a nationwide database.

## Methods

### Data resource and study population

The public medical insurance system in South Korea, known as the National Health Insurance Service, covers approximately 98% of the South Korean population as South Korean law requires every resident to be covered by this scheme^15,16^. Under this national insurance system, the Health Insurance Review and Assessment Service (HIRA) reviews and evaluates medical costs and healthcare service quality. Currently, HIRA provides nationwide claims data, which are collected during this process for researchers. HIRA claims data include age, sex, diagnoses, medical costs claimed, procedures, prescribed drugs, and a unique anonymous number for each patient^16^. The strength of HIRA data lies in the fact that the National Health Insurance Service covers the entire South Korean population which is ethnically homogeneous. Many population-based studies have been conducted based on HIRA data^17–19^.

This is a population-based case-control study nested in a retrospective cohort using HIRA claims data collected between 2007 and 2016. Patients and controls were identified based on the Korean Classification of Disease (KCD), which is an adapted version of International Classification of Disease (ICD). In the present study, we used the KCD diagnosis code G61.8 (other inflammatory polyneuropathies) to identify patients with CIN. Currently, the KCD format does not provide a diagnosis code solely for CIDP or MMN and these diagnoses are incorporated in the diagnosis of other inflammatory polyneuropathies (G61.8). For the robustness of the data, an operational definition of CIN was made based on the diagnosis code and the following criteria. After excluding patients with a pre-existing G61.8 diagnosis in 2007, we included patients with the following criteria: (1) age ≥18 years, (2) ≥2 G61.8 claims reported at least 90 days apart, (3) history of inpatient hospitalization or outpatient visit at the department of neurology or rehabilitation medicine, and (4) underwent nerve conduction studies. The inclusion criterion requiring ≥2 claims reported at least 90 days apart was based on a previous epidemiologic study and the diagnostic criteria of CIDP and MMN that require the disease duration to be ≥2 months and ≥1 month, respectively^20–22^. We excluded the following people: (1) patients with a secondary cause of polyneuropathy that should be differentiated from CIN, including hereditary demyelinating neuropathy (G60.0), monoclonal gammopathy (D47.2), polyneuropathy, organomegaly, endocrinopathy, monoclonal gammopathy, skin change syndrome (G62.9, D47.7), drug or toxin-induced neuropathy (G62), *Borrelia burgdorferi* infection (A69.2), diphtheria (A36.8), and amyloidosis (E85) and (2) patients with a diagnosis of osteoporosis before the initial diagnosis of CIN. For the control group, we identified patients aged ≥18 years whose claims included the diagnosis code of Z00.0 (general medical examination). Of these patients, those with a previous diagnosis of osteoporosis, a diagnosis code of G61.8, and neurologic disease that could be differentiated from CIN were excluded. The utilization of and access to the HIRA database was approved by HIRA. The Severance Hospital Institutional Review Board approved this research (approval no. 4-2017-0318) and the study was conducted in accordance with the Declaration of Helsinki. Informed consent was waived due to the retrospective nature of the study.

### Data collection

The primary outcome of this study was a new diagnosis of osteoporosis. Such diagnoses are made and entered by physicians at each site based on their clinical impression. HIRA provides guidelines regarding the clinical indications for conducting dual energy x-ray absorptiometry and prescribing medication for osteoporosis. It also reviews whether payment claims correctly follow these guidelines. Previous studies demonstrated a concordance between the HIRA database and actual diagnoses made using clinical information^23,24^. Data regarding the basic demographics (age and sex), past medical history, and Charlson comorbidity index (CCI) score were collected. CCI is the most widely used comorbidity index; it categorizes comorbidities of patients based on the ICD codes and calculates a comorbidity score by summing the score of each category weighted according to the adjusted risk of mortality. A higher CCI score indicates greater comorbidities^25^. In the current study, CCI score was measured according to the algorithms suggested by Jang *et al*., which is based on the algorithm of Quan *et al*.^24,26^. The use of corticosteroids, intravenous immunoglobulin, immunosuppressants, or immunomodulatory drugs (alemtuzumab, azathioprine, cyclophosphamide, cyclosporine, etanercept, interferon, mycophenolate mofetil, methotrexate, and rituximab) were recorded. In South Korea, intravenous immunoglobulin obtained reimbursement approval for use in patients with CIDP in June 2015 and in the patients with MMN in April 2018. As medical service providers have processed claims for insurance benefits since this time point, the intravenous immunoglobulin prescription data before this point or unclaimed prescriptions after this point were not recorded in the HIRA database and could not be analyzed in the present study. Corticosteroid doses were converted to the prednisone equivalent dose, and the average daily dose of corticosteroid was calculated by dividing the cumulative prednisone dose by the duration of treatment. Pulsed dexamethasone treatment was defined as the prescription of ≥40 mg dexamethasone for ≥4 consecutive days and pulsed methylprednisolone treatment was defined as the prescription of ≥500 mg methylprednisolone for ≥4 consecutive days^27,28^. The annual incidence and prevalence was calculated by using patients of all ages. The annual incidence and prevalence of CIN was calculated by dividing the number of newly diagnosed patients with CIN and the number of the patients who visited healthcare facilities with a diagnosis of CIN by the population of each year, respectively. The total population of South Korea was obtained from the Korean Statistical Information Service (KOSIS; http://kosis.kr/), which provides official statistics for South Korea.

### Propensity score matching

Osteoporosis is associated with age and sex^29^. Therefore, a propensity score matching technique was adopted to attenuate the compounding effects of age, sex, index year, and CCI score between patients with CIN and controls. The propensity score was calculated using a logit model for matching variables (age, sex, index year, and CCI score) by considering patients with CIN as the treatment group and predicting probabilities. A 1:1 propensity score matching was fulfilled to match patients with CIN with controls within a caliper of 0.05. Propensity score matching revealed that there were no significant differences in age, sex, and CCI score between the groups. A total of 1170 participants (585 patients with CIN and 585 controls) were used in the final analysis.

### Statistical analysis

The results are presented as numbers (percentages) for categorical variables and median [Q1–Q3] for continuous variables as these did not meet the normality assumption. For data before propensity score matching, the Chi-square test and Mann–Whitney U test were used for the comparison of categorical variables and continuous variables between the 2 groups, respectively. For data after propensity score matching, the McNemar test and the Wilcoxon signed rank test were used for the comparison of categorical variables and continuous variables between the 2 groups, respectively. We used a stratified Cox proportional hazards regression model to estimate the effect of CIN on the development of osteoporosis when taking the matching into account. Kaplan–Meier analysis was performed to analyze the cumulative incidence curve of osteoporosis between patients with CIN and controls. The log-rank test was performed to assess the difference in the incidence curves between the 2 groups. All p-values were two-sided and a p value < 0.05 was considered statistically significant. SAS 9.4 software (SAS Institute, Cary, NC) was used for the statistical analysis. R software (version 3.4.0, R Foundation for Statistical Computing, Vienna, Austria) was used to draw the cumulative incidence curves.

## Results

### Baseline demographics and clinical features of CIN and control groups

**Figure 1:**
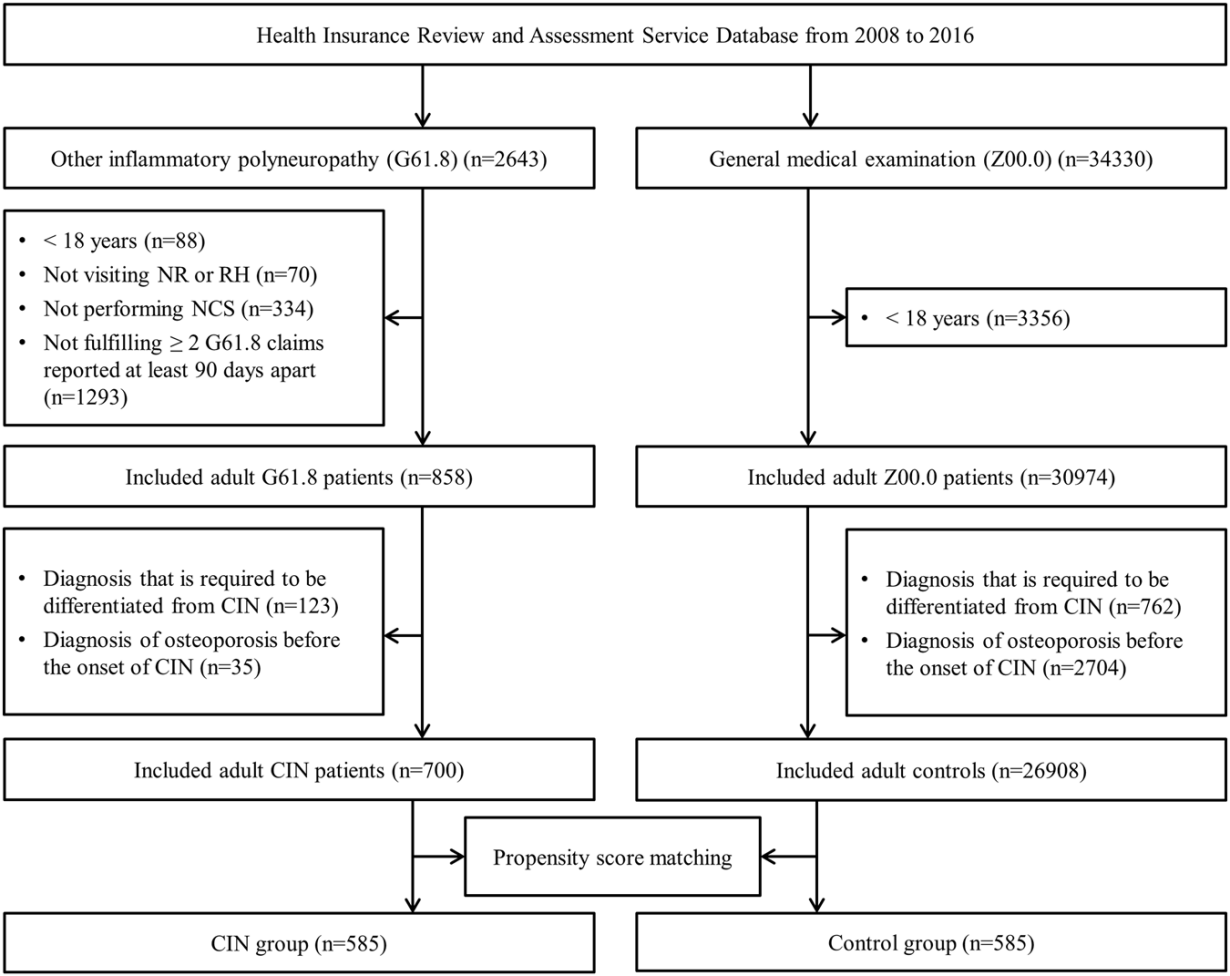
Flowchart of case inclusion process. NR, department of neurology; RH, department of rehabilitation medicine; NCS, nerve conduction study; CIN, chronic inflammatory neuropathy.

After excluding patients with pre-existing CIN in 2007, a total of 2643 patients with a diagnosis code of other inflammatory polyneuropathy (G61.8) were identified between 2008 and 2016. Of these, 858 fulfilled the inclusion criteria. The exclusion criteria applied to a further 158 patients. Finally, 700 patients who satisfied the operational definition of CIN were included in the study (Fig. 1). The annual incidence of CIN was 0.2 per 100,000 per year and prevalence rate was 0.6 per 100,000 population. Baseline demographic and clinical characteristics of patients with CIN and controls are presented in Table 1. There were 427 (61.0%) male and 273 (39.0%) female participants with CIN with a male to female ratio of 1.6:1. There was significant difference in terms of sex (p < 0.001) and CCI score (p < 0.001) between the CIN and control groups before propensity score matching. Using propensity score matching, 585 patients with CIN and 585 controls were finally used in the analysis and it was confirmed that there were no significant differences in age, sex, and CCI scores between the 2 groups.

**Table 1:** Baseline demographic and clinical characteristics of patients with chronic inflammatory neuropathy and controls before and after propensity score matching.

	Before matching			After matching		
	CIN	Control	P-value	CIN	Control	P-value
	(n = 700)	(n = 26908)		(n = 585)	(n = 585)	
Age, years	55.0 [44.0–66.0]	56.0 [45.0–68.0]	0.060	55.0 [43.0–66.0]	56.0 [43.0–68.0]	0.139
Sex			<0.001			0.882
Women	273 (39.0)	13321 (49.5)		235 (40.2)	233 (39.8)	
Men	427 (61.0)	13587 (50.5)		350 (59.8)	352 (60.2)	
CCI score			<0.001			0. 780
0	132 (18.9)	8347 (31.0)		132 (22.6)	132 (22.6)	
1	137 (19.6)	6218 (23.1)		120 (20.5)	130 (22.2)	
≥2	431 (61.6)	12343 (45.9)		333 (56.9)	323 (55.2)	
Median CCI score	2.0 [1.0–4.0]	1.0 [0–3.0]	<0.001	2.0 [1.0–4.0]	2.0 [1.0–4.0]	0.895
Treatment						
IVIG*	31 (4.4)			29 (5.0)		
Corticosteroid	600 (85.7)			501 (85.6)		
Immunosuppressant	304 (43.4)			253 (43.3)		

*Intravenous immunoglobulins that have been prescribed and claimed since June 2015.

Data are expressed as count (percentage) or median [Q1–Q3] values.

CCI, Charlson comorbidity index; CIN, chronic inflammatory neuropathy; IVIG, intravenous immunoglobulin.

### Incidence of osteoporosis in CIN and control groups

**Figure 2:**
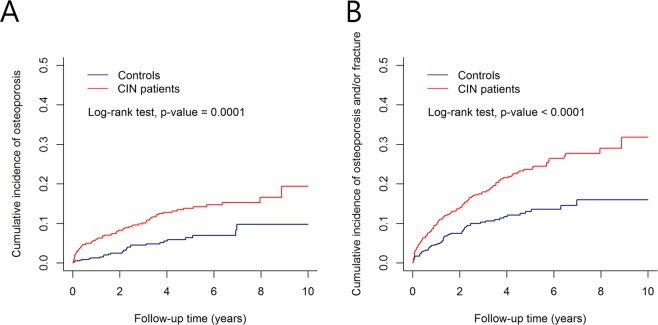
Cumulative incidence curves: (**A**) osteoporosis and (**B**) osteoporosis and/or fracture for chronic inflammatory neuropathy (CIN) and control groups.

Cumulative incidences of 1) osteoporosis and 2) osteoporosis and/or any fracture for the CIN group and control group are shown in Fig. 2. The cumulative incidences of osteoporosis and osteoporosis/fracture were significantly higher among patients with CIN than among controls. Patients with CIN had a 2.293-fold (hazard ratio [HR] = 2.293, 95% confidence interval [CI] 1.460–3.601) increased risk of developing osteoporosis compared with controls (Table 2). Patients with CIN also had an increased risk of developing any fracture (HR = 1.753, 95% CI 1.178–2.608) and osteoporosis and/or fracture (HR = 1.943, 95% CI 1.411–2.678). We further evaluated the risk of osteoporosis in subgroups of patients stratified by age and sex. In the sex-specific analysis, osteoporosis occurred in 10% of male patients with CIN and 15.7% of female patients with CIN. The risk of osteoporosis was significantly higher among male patients with CIN than among male controls (HR = 5.404, 95% CI 2.252–12.969). Conversely, there were no significant differences in the risk of osteoporosis between female patients with CIN and female controls.

**Table 2:** Hazard ratios for osteoporosis and/or fracture in patients with chronic inflammatory neuropathy and the risk of osteoporosis stratified by age and sex.

Characteristics	CIN		Control		CIN vs. control		
	Total	Case (%)	Total	Case (%)	HR	95% CI	P-value
Outcomes							
Osteoporosis	585	72 (12.3)	585	28 (4.8)	2.293	1.460–3.601	<0.001
Fracture	585	74 (12.6)	585	37 (6.3)	1.753	1.178–2.608	0.006
Osteoporosis or fracture	585	124 (21.2)	585	58 (9.9)	1.943	1.411–2.678	<0.001
**Occurrence of osteoporosis**							
Sex							
Men	350	35 (10.0)	352	6 (1.7)	5.404	2.252–12.969	<0.001
Women	235	37 (15.7)	233	22 (9.4)	1.41	0.809–2.459	0.225
Age, years							
18–49	228	22 (9.7)	213	5 (2.4)	3.684	1.375–9.873	0.010
≥50	357	50 (14.0)	372	23 (6.2)	2.027	1.211–3.391	0.007

Data are expressed as count (percentage) values.

CIN, chronic inflammatory neuropathy; HR, hazard ratio; CI, confidence interval.

### Comparison between male and female patients with CIN

We compared the clinical factors between male and female patients with CIN to evaluate the possible factors that may have affected the high risk of osteoporosis among male patients with CIN (Table 3). Male patients with CIN were older than female patients with CIN (p = 0.022) and had a higher CCI score (p = 0.006). With regard to comorbidities, the prevalence of diabetes mellitus, liver disease, and cerebrovascular disease was higher among male patients with CIN than among female patients (p = 0.020, p = 0.003 and p = 0.003, respectively). There were no significant differences in the use of corticosteroids and immunosuppressive or immunomodulating agents between male and female patients.

**Table 3:** Comparison of clinical and treatment-related features between male and female patients with chronic inflammatory neuropathy.

	Male (n = 350)	Female (n = 235)	P-value
Age, years	56.0 [45.0–66.0]	53.0 [39.0–64.0]	0.022
CCI score			0.013
0	73 (20.9)	59 (25.1)	
1	61 (17.4)	59 (25.1)	
≥2	216 (61.7)	117 (49.8)	
Median CCI score	2.0 [1.0–4.0]	1.0 [0–3.0]	0.006
Past medical history			
Connective tissue disease	15 (4.3)	18 (7.7)	0.083
Diabetes mellitus	148 (42.3)	77 (32.8)	0.020
Renal disease	32 (9.1)	12 (5.1)	0.070
Liver disease	84 (24.0)	33 (14.0)	0.003
Cerebrovascular disease	84 (24.0)	33 (14.0)	0.003
Cancer	47 (13.4)	21 (8.9)	0.097
Metastatic cancer	9 (2.6)	6 (2.6)	0.989
Number of patients using corticosteroids	306 (87.4)	195 (83.0)	0.132
Average daily dose ≤ 12 m from onset	17.5 [7.5–31.3]	15.8 [7.6–31.5]	0.478
Number of patients using IVIG*	13 (3.7)	16 (6.8)	0.091
Number of patients using immunosuppressant	151 (43.1)	102 (43.4)	0.950

*Intravenous immunoglobulins that have been prescribed and claimed since June 2015.

Data are expressed as count (percentage) or median [Q1–Q3] values.

CCI, Charlson comorbidity index; IVIG, intravenous immunoglobulin.

### Comparison between patients with CIN who developed osteoporosis and those who did not

We compared clinical factors between 72 patients with CIN who developed osteoporosis (osteoporosis group) and 513 patients with CIN who did not (non-osteoporosis group). The results are shown in Table 4. The proportion of female patients was significantly higher in the osteoporosis group than in the non-osteoporosis group (p = 0.038). Regarding treatment, the average daily dose of corticosteroids within 12 months from CIN diagnosis was significantly higher in the osteoporosis group than in the non-osteoporosis group (p = 0.001). The difference was found to be significant in terms of the average daily dose of oral prednisolone (p < 0.001), whereas no difference was observed in the dose of pulsed methylprednisolone (p = 0.842). Conversely, the proportion of patients who received intravenous immunoglobulin or immunosuppressive agents was significantly lower in the osteoporosis group than in the non-osteoporosis group (p = 0.038 and p = 0.020, respectively). In terms of preventive therapy, 38.9% of patients with osteoporosis and 23.4% of patients without osteoporosis were treated with vitamin D and/or calcium.

**Table 4:** Comparison of clinical and treatment-related feature between patients with chronic inflammatory neuropathy who developed osteoporosis and those who did not.

	Osteoporosis	Non-osteoporosis	P-value
	(n = 72)	(n = 513)	
Age, years	57.0 [44.0–64.0]	54.0 [43.0–66.0]	0.378
Sex			0.038
Female	37 (51.4)	198 (38.6)	
Male	35 (48.6)	315 (61.4)	
Median CCI score	2.0 [0–3.0]	2.0 [1.0–4.0]	0.479
Past medical history			
Connective tissue disease	5 (6.9)	28 (5.5)	0.585
Diabetes mellitus	26 (36.1)	199 (38.8)	0.662
Renal disease	2 (2.8)	42 (8.2)	0.103
Liver disease	11 (15.3)	106 (20.7)	0.285
Cerebrovascular disease	16 (22.2)	101 (19.7)	0.615
Cancer	10 (13.9)	58 (11.3)	0.522
Metastatic cancer	3 (4.2)	12 (2.3)	0.413
Number of patients using corticosteroids	64 (88.9)	437 (85.2)	0.401
Average daily dose ≤12 m from onset	19.6 [10.8–49.3]	16.2 [7.2–29.1]	0.001
Pulsed methylprednisolone during follow-up	22 (30.6)	178 (34.7)	0.488
Total dose, mg	6250.0 [3750.0–6250.0]	6250.0 [3750.0–6250.0]	0.842
Oral prednisolone during follow-up	61 (84.7)	404 (78.8)	0.240
Average daily dose, mg	14.6 [8.7–31.8]	4.9 [1.8–10.0]	<0.001
Number of patients using IVIG*	0 (0)	29 (5.7)	0.038
Number of patients using immunosuppressant	22 (30.6)	231 (45.0)	0.020
Azathioprine	18 (25.0)	191 (37.2)	0.043
Cyclosporine	0 (0)	24 (4.7)	0.060
Cyclophosphamide	2 (2.8)	25 (4.9)	0.561
Mycophenolate mofetil	3 (4.2)	43 (8.4)	0.213
Methotrexate	1 (1.4)	9 (1.8)	>0.999

*Intravenous immunoglobulins that have been prescribed and claimed since June 2015.

Data are expressed as count (percentage) or median [Q1–Q3] values.

CCI, Charlson comorbidity index; IVIG, intravenous immunoglobulin.

## Discussion

The present study revealed a high risk of osteoporosis among patients with CIN based on nationwide cohort. The results showed that patients with CIN had a 2.293-fold increased risk of osteoporosis compared with matched controls. Although osteoporosis is frequent among women in general, the risk of osteoporosis was significantly higher among male patients with CIN than among male controls. These results highlight the importance of early screening to detect osteoporosis in both male and female patients with CIN. In addition, patients with CIN who developed osteoporosis received higher doses of corticosteroids than controls, which may suggest that high dose corticosteroid use is one of the risk factors for osteoporosis among patients with CIN.

Only a few studies have evaluated the occurrence of osteoporosis among patients with CIN, with varying results^7,9,30^. One study evaluated bone mineral density in 9 patients with CIDP during pulsed steroid treatment and 5 (55.6%) patients developed osteoporosis over 3 years^9^. Our recent retrospective study also demonstrated that 39.0% of CIDP patients had a bone mineral density in the osteoporotic range^7^. In contrast, another study that measured bone mineral density during pulsed steroid treatment in 40 patients with CIDP reported that none of the patients developed osteoporosis after 12 months^30^. Previous results are based on small study populations with a short follow-up duration and it is difficult to determine whether patients with CIN have an increased risk of osteoporosis compared with those without CIN. Therefore, a population-based study with an adequate follow-up period is necessary to assess the risk of osteoporosis for this relatively uncommon disease.

The high incidence of osteoporosis among patients with CIN may result from prolonged use of corticosteroids, which causes bone loss by inhibiting osteoblast function and increasing osteoclast formation^31,32^. In the present analysis, patients with CIN who developed osteoporosis received higher doses of steroids than those who did not. In addition, the difference was attributed to the difference in the dose of oral prednisolone but not to the dose of intravenous pulsed steroids. This is consistent with previous reports showing that the cumulative steroid dose is inversely correlated with bone mineral density and that prolonged oral steroid treatment is more deleterious to bone density than pulsed intravenous administration^31,33,34^. In contrast, patients who did not develop osteoporosis were more likely to have undergone treatment with immunosuppressive agents or intravenous immunoglobulin. The steroid-sparing effect of these alternative treatments may have reduced the risk of osteoporosis, although further studies are required to investigate this.

There are some other possible explanations for the high incidence of osteoporosis in patients with CIN. First, functional disability resulting from CIN may have an adverse effect on bone density. Previous studies showed that 39.5% of patients with CIDP had a functional status of chair-bound or worse at the time of initial treatment^5^, and more than half of the patients with MMN displayed abnormality in gait before treatment^6^. This disability can result in decreased muscle mass, lack of physical activity, and low sunlight exposure, all of which are known risk factors for bone loss^35–37^. Concordantly, our recent study revealed that osteoporosis among patients with CIDP was associated with impaired functional status^7^. Second, CIN itself can cause bone loss. Previous studies have revealed an increased risk of osteoporosis among patients with autoimmune neurological diseases^38–40^. Chronic inflammation in these diseases is thought to promote bone loss through pro-inflammatory cytokines by inhibiting osteoblast activity and promoting osteoclast activity^41,42^. Interleukin-17 is one of the proinflammatory cytokines that contributes to bone loss^43^, and interleukin-17 levels were found to be high in both active CIDP patients and in an animal model of CIDP^44,45^. In addition, tumor necrosis factor alpha (TNF-α), which is known to induce osteoclastogenesis, plays a main role in animal models of autoimmune neuropathy, and autoimmune neuritis was attenuated in TNF-α knockout mice, probably by altering the balance between proinflammatory and anti-inflammatory macrophages^46–48^.

It is interesting to note that the risk of osteoporosis was especially high among male patients with CIN. In general, women have a significantly higher risk of osteoporosis than men, and the rate of osteoporosis among women aged ≥50 years is nearly 4 times higher than that among men^29^. Thus, although the absolute proportion of patients with CIN with newly developed osteoporosis was higher among women than among men, the relative risk was not significantly different from that among controls because of the high baseline risk among the general female population. In contrast, the risk of osteoporosis among male patients with CIN was significantly higher than that among controls. This may reflect both a low incidence of osteoporosis among the general male population and an increased risk of osteoporosis among male patients with CIN. Overall, the male to female ratio of osteoporosis occurrence in patients with CIN was not as markedly different as in controls. This finding may imply that the development of osteoporosis in both male and female patients with CIN is attributable to a common factor: this could be CIN itself, disability from the disease, or related treatments.

The high risk of osteoporosis or fracture in male patients with CIN may be partially due to an older age or a higher proportion of diabetes mellitus among male patients with CIN compared with female patients. Previous studies revealed that male patients with CIDP were older than female patients with CIDP. One study reported that the prevalence of CIDP was higher among male than female patients, particularly those over the age of 55 years^12^. Other studies showed that the prevalence of CIDP continuously increased after the age of 60–70 years among male patients with CIDP, while the prevalence started to decrease from this point in female patients^11,13^. The risk of osteoporosis increases with age^29^ and older age in male patients with CIN may further increase the risk of osteoporosis. In addition, diabetes mellitus was observed more frequently among male patients with CIN than among female patients, which is consistent with the previous studies^49,50^. Patients with type 1 diabetes mellitus have decreased bone mineral density^51,52^, and the risk of fracture is increased among patients with type 2 diabetes mellitus^53^. Thus, the higher incidence of fracture may result from the higher proportion of diabetes mellitus among male patients with CIN.

Despite a high incidence of osteoporosis among patients with CIN, only 38.9% of patients were treated with Vitamin D and/or calcium. Recent guidelines recommend that all adults taking ≥2.5 mg/day of prednisone for more than 3 months should take optimal doses of calcium and vitamin D^54^. However, not enough attention is paid to the risk of osteoporosis in patients taking corticosteroids. Hougardy *et al*. showed that only 31% of those taking oral corticosteroids over a prolonged period were given medication to prevent osteoporosis^55^. A previous survey conducted by the Korea Centers for Disease Control & Prevention similarly showed that less than half of women over 50 diagnosed with osteoporosis were being treated^56^. Lack of awareness has been similarly observed among neurologists. In previous studies regarding bone health in patients with myasthenia gravis, only half of patients undergoing corticosteroid treatment were treated with bisphosphonates^57,58^. In addition, previous studies show that the physician diagnosis rate and treatment rate of osteoporosis is lower for male patients than female patients^55,59^. Thus, the current study may contribute to raising awareness among neurologists regarding bone loss in patients with CIN, especially in male patients.

Some limitations to this study should be acknowledged. First, the results are based on the HIRA data, which are obtained for the purposes of administrative billing. We were unable to assess risk factors for osteoporosis that are not collected in the HIRA database. Second, data regarding the clinical features, laboratory results, disease course and severity, and results of nerve conduction studies, as well as bone mineral density and biochemical parameters, are not provided in the HIRA data. Therefore, the diagnosis of CIN and osteoporosis had to rely upon the KCD diagnosis code and operational definition. However, the demographic characteristics of patients with CIN in the present study, including age, gender ratio, and annual incidence, correlates well with those reported in previous studies^10–13,60^, which may imply that the diagnosis is reliable. In addition, previous reports demonstrated a considerable correlation between prevalence calculated based on the HIRA database and actual diagnoses made using clinical data^23,24^. Third, the use of prophylactic therapy with vitamin D or calcium could not be adequately analyzed. In contrast to the other medications, including steroids and immunosuppressive agents, calcium and vitamin D supplements are available to the patients without prescription. As the current study is based on the data obtained for the purposes of administrative billing, the use of medications without prescription could not be analyzed.

In conclusion, this nationwide, population-based study showed that the risk of osteoporosis among patients with CIN is significantly higher than among matched controls. The risk was particularly high among male patients with CIN, who are usually perceived to have a lower risk of osteoporosis than female patients. Moreover, it was found that not enough patients were taking preventive treatment for osteoporosis. The result highlights the importance of early screening for osteoporosis and initiating (or switching) pharmacologic treatment for osteoporosis in patients with CIN.

## Data Availability

Data are available at Healthcare Bigdata Hub (http://opendata.hira.or.kr) after the qualification process and approval from HIRA for the utilization of and access to the HIRA database.
